# Systematic Analysis of Stability Patterns in Plant Primary Metabolism

**DOI:** 10.1371/journal.pone.0034686

**Published:** 2012-04-13

**Authors:** Dorothee Girbig, Sergio Grimbs, Joachim Selbig

**Affiliations:** 1 Max-Planck Institute for Molecular Plant Physiology, Potsdam, Germany; 2 Institute of Biochemistry and Biology, University of Potsdam, Potsdam, Germany; Glasgow University, United Kingdom

## Abstract

Metabolic networks are characterized by complex interactions and regulatory mechanisms between many individual components. These interactions determine whether a steady state is stable to perturbations. Structural kinetic modeling (SKM) is a framework to analyze the stability of metabolic steady states that allows the study of the system Jacobian without requiring detailed knowledge about individual rate equations. Stability criteria can be derived by generating a large number of structural kinetic models (SK-models) with randomly sampled parameter sets and evaluating the resulting Jacobian matrices. Until now, SKM experiments applied univariate tests to detect the network components with the largest influence on stability. In this work, we present an extended SKM approach relying on supervised machine learning to detect patterns of enzyme-metabolite interactions that act together in an orchestrated manner to ensure stability. We demonstrate its application on a detailed SK-model of the Calvin-Benson cycle and connected pathways. The identified stability patterns are highly complex reflecting that changes in dynamic properties depend on concerted interactions between several network components. In total, we find more patterns that reliably ensure stability than patterns ensuring instability. This shows that the design of this system is strongly targeted towards maintaining stability. We also investigate the effect of allosteric regulators revealing that the tendency to stability is significantly increased by including experimentally determined regulatory mechanisms that have not yet been integrated into existing kinetic models.

## Introduction

Understanding the way in which individual components interact in a biological network is a major goal of systems biology [1]. The prediction of a system's response to internal or external perturbations, as well as the identification of components that play a major role in this response, requires mathematical modeling [2]. Approaches for mathematical modeling of metabolic networks can be subdivided into (1) structural modeling and (2) kinetic modeling. Structural modeling relies solely on information about the network structure (stoichiometry) and enables the analysis of system properties in a steady state. In contrast, kinetic modeling allows the analysis of the dynamic properties of the network and is not restricted to steady states. However, this approach relies on detailed knowledge about all enzymatic rate laws and kinetic parameters in the system, which are often difficult to obtain experimentally.

Structural kinetic modeling (SKM) combines principles from both approaches and offers a powerful tool to analyze the local dynamic properties of metabolic networks in a steady state [3]. This restriction to steady state scenarios allows the method to rely on less prior knowledge than would be required for the construction of a comprehensive kinetic model. In kinetic models, the dynamic properties of a steady state can be derived from the eigenvalues of its Jacobian matrix. This matrix contains the partial derivatives of the reaction rates, and therefore its computation requires detailed knowledge about the kinetic rate laws, as well as their kinetic parameters. The basic idea of SKM is the construction of a parameterized version of the Jacobian matrix of a system in a steady state, in which the model parameters encode information about the enzyme-metabolite interactions, avoiding the necessity to compute partial derivatives. Consequently, instead of relying on a detailed set of rate equations, together with accurate estimates of the kinetic parameters, the Jacobian matrix then depends only on a set of SK-model parameters.

In mathematical terms, the SK-model parameters are partial derivatives of the rate equations in a system that has been normalized to represent a particular steady state. Thus, the parameters describe the influence of changes in metabolite concentrations on the reaction rates in this steady state. In enzymatic reactions, this influence depends largely on the amount of saturation of an enzyme with its metabolites. Experimental values for these parameters are often unknown in practice. However, SKM enables the systematic analysis of a steady state's dynamic properties by using a Monte Carlo approach. This approach comprises (1) the generation of a large number of parameter sets by sampling them from predefined intervals, (2) the construction of the corresponding Jacobian matrices, and (3) the evaluation of these matrices based on their eigenvalues. The statistical exploration of the parameter space can then indicate regions associated with different local properties of the system. Because the model parameters offer a straight-forward biological interpretation, they enable the identification of the enzymes and metabolites that play major roles in determining the system's behavior.

One system property of particular interest is local stability, which can be understood as the robustness of a steady state to perturbations. A stable steady state allows the fine-tuned response of the reaction rates to perturbations, eventually enabling the return to the original steady state. In mathematical terms, a steady state is stable if the largest real part of the Jacobian matrix's eigenvalues is negative.

**Figure 1:**
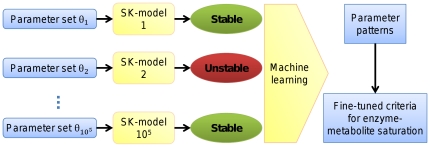
Workflow for Monte Carlo based model generation and the subsequent detection of patterns by decision trees. First, a large number of SK-models is created based on randomly sampled parameter sets. They allow the detection of those parameter sets that lead to a stable or unstable steady state, respectively. Using the model parameters as feature vectors and the stability information as class labels, a classifier can learn those patterns in the parameter space with highest discriminatory power between both classes. These patterns then describe quantitative criteria for the degree of saturation of individual enzymes in the pathway that ensure stability or instability.

So far, SKM experiments that searched for stability conditions have focused on the detection of individual enzymes to identify single important reactions [4], [5]. However, specific changes in flux distributions can generally be caused by more than one enzyme in a pathway [6], or sometimes even require different enzymes acting together in an orchestrated manner [7], [8]. Here we extend this existing approach by demonstrating how SKM enables the detection of ensembles of enzymes or metabolites that act together in an orchestrated manner to control fluxes through the pathway. As shown in Figure 1, we use the information about stable and unstable states as class labels, and train classifiers to detect parameter regions associated with stability and instability. To this end, we use decision trees to search for patterns in the model parameter space, and to derive quantitative thresholds for the parameters.

We demonstrate the application of our extended SKM approach to a detailed model of the autocatalytic Calvin-Benson cycle (CBC), which is the main pathway in plant cells for the fixation of atmospheric 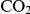 to produce energy-rich biomolecules. Enzyme kinetics and regulatory mechanisms of the CBC have been intensely studied *in vivo* and *in silico*. This has led to the development of a large number of kinetic models to simulate and explain the processes underlying the CBC and related pathways. Nevertheless, there has been an ongoing debate about the accuracy and explanatory power of these models [9], [10]. The importance of this issue was also demonstrated in a recent study that introduced a systematic approach to solve controversies about the possible numbers of steady states, revealing strong dependencies of predicted system properties on the kinetics used in a model [11]. This emphasizes the importance of a modeling framework like SKM, that is flexible with respect to kinetic rate laws and the corresponding kinetic parameters, and that allows the systematic identification of conditions related to distinct system behaviors.

In a recent study, SKM was applied to investigate the stability of metabolic cycles like the CBC, with a special focus on autocatalytic architectures [12]. The investigation of a simple autocatalytic system revealed a general tendency towards stability. Furthermore, the impact of single model parameters on stability was systematically assessed, and stability conditions for specific parameter combinations were derived analytically. An SKM-based analysis of a simplified CBC model, without any regulatory interactions, was conducted in order to demonstrate how this method can assist in detecting parameter regions associated with stability [3]. However, this model relied on simplifying assumptions such as fixed global values for all model parameters. Furthermore, it did not take into account the connections of the CBC to adjacent pathways in the cytosol, as well as the regulatory mechanisms that are required to finely tune the interactions between the CBC and these connected pathways [13]–[15].

In this work, we analyze an extended model of the autocatalytic Calvin-Benson cycle (CBC) including allosterically regulated starch and sucrose synthesis, adenosine triphosphate (ATP) production, and an entry point to cytosolic amino acid metabolism. Using this system as an example, we show how SKM can help in systematically assessing the influence of single rate equations in the CBC by enabling quick alterations in model structure, and by directly monitoring the evoked effect on the system's properties. For example, different implementations of transporter-associated rate laws can cause fundamental alterations in the dynamic properties of the system. Our results confirm that stability is highly prioritized in the design of the system, and that allosteric regulation can increase the chance for stability significantly. We also demonstrate the limitations of existing kinetic models in assessing the role of newly detected regulatory mechanisms and we show how such interactions can easily be included into an SK-model without the necessity of knowing the corresponding kinetic parameters or rate equations.

Using our new machine learning approach we can confirm the importance of ‘key enzymes’ like ribulose 5-phosphate kinase and ATP phosphohydrolase (ATPase) to ensure stability. Reliable patterns ensuring stability are more frequently detected than patterns that guarantee unstable steady states, again emphasizing that local stability is largely favoured over instability in the investigated pathway.

## Results

### Construction of structural kinetic models for the Calvin-Benson cycle

**Figure 2:**
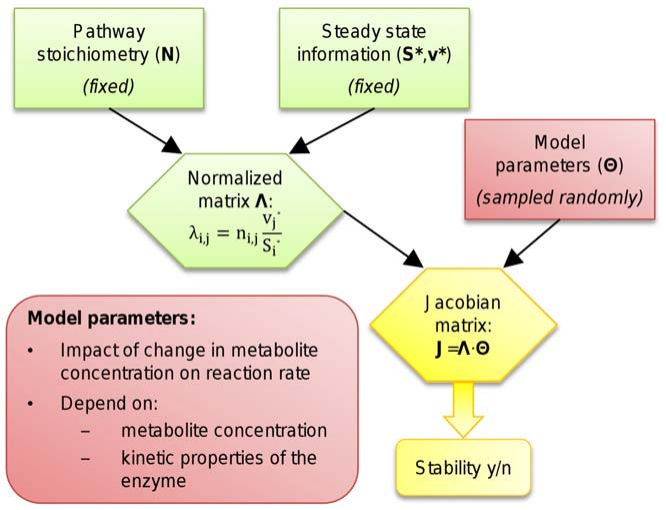
The principles of structural kinetic modeling. Normalization of the pathway-specific stoichiometric matrix 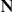 with respect to steady state concentrations 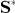 and fluxes 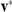 produces the normalized matrix 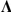. Together with the model parameters in the matrix 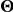, it uniquely defines the Jacobian matrix of the system in the steady state. Evaluation of the eigenvalues of the Jacobian matrix then indicates whether the steady state is stable.

As shown in Figure 2, construction of an SK-model requires knowledge about the network's stoichiometry, as well as the concentrations and fluxes that characterize the steady state of interest. In principle, these values can be derived experimentally without requiring detailed knowledge about rate laws or kinetic parameters. However, the method crucially relies on the fulfillment of the steady-state assumption that there are zero net changes in concentrations over time. Therefore, when studying a specific biological system for which a kinetic model is available, the calculation of concentrations and fluxes is generally assisted by numerical simulation or optimization techniques to reach sufficient numerical accuracy [14], [16].

We used the kinetic model by Laisk et al. (2009) [17] as a reference for network stoichiometry and steady-state information. This model was chosen, because a recent study by Arnold and Nikoloski (2011) [10] based on a slightly smaller predecessor model [18] showed that its predicted steady state agrees well with experimental measurements. It includes the reactions of the CBC, starch and sucrose metabolism, ATP and reduced nicotinamide adenine dinucleotide phosphate (NADPH) generation, parts of the cytosolic glycolysis and gluconeogenesis pathway, and an entry point to amino acid metabolism via alanine production. In total, we incorporated 35 metabolites and 29 reactions, occurring across two compartments (chloroplast stroma and cytosol), as well as nine allosteric regulators. The enzyme-metabolite relationships were represented by 87 model parameters.

**Figure 3:**
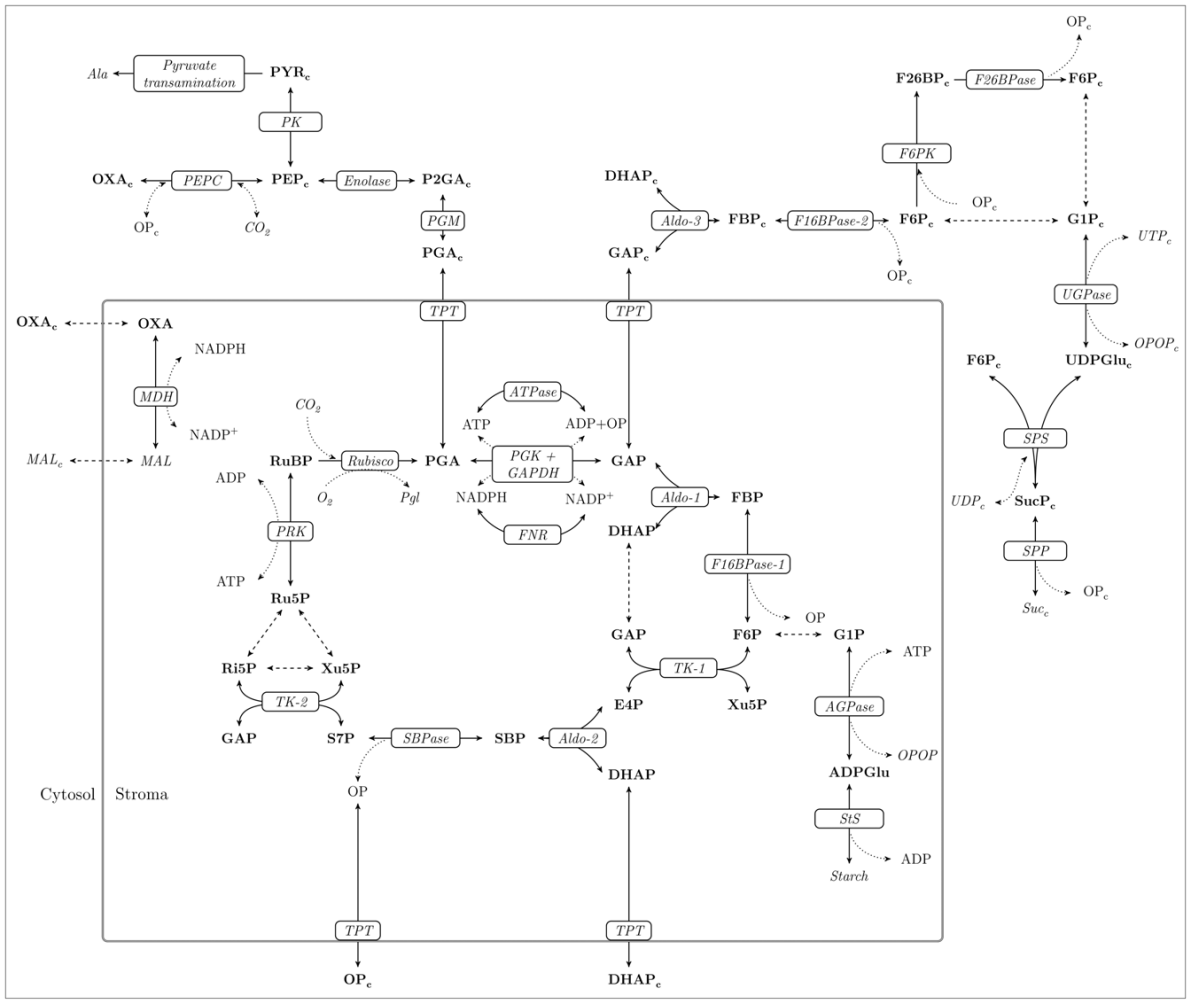
Network underlying the SK-model of the CBC and related pathways. Compounds written in italics represent external substances the concentrations of which are kept constant in the model. Dotted lines indicate the reactions of cofactors. Dashed lines connect metabolites that are assumed to be in equilibrium so that their concentration changes are directly proportional to each other. The proportions of the individual concentrations of these metabolites then depend solely on their equilibrium constants.

As steady state concentrations, we used the experimental values provided by the authors of the kinetic model. However, we refined these values by simulation until they fulfilled the steady state assumption with sufficient numerical accuracy (see section Methods for details). Although included in the original model, we omitted the photosynthetic electron transfer chain, assuming constant photosynthetic regeneration of ATP and NADPH. A schematic overview of the reactions and metabolites included in the model is given in Figure 3. All metabolite and enzyme abbreviations are explained in Table S1.

### The role of membrane transport regulation

The triose-phosphate translocator (TPT) exchanges triose-phosphates and 3-phosphoglycerate (PGA) synthesized by the CBC with inorganic orthophosphate (Pi) from the cytosol via an antiport mechanism. Sustained phosphate supply is important to maintain constant flux through the pathway. Consequently, changes in transport rates can have a high impact on stromal concentrations, making the TPT a bottleneck for the system's ability to maintain stability [19]. To model TPT mediated transport an ensemble of regulatory interactions was included in the kinetic model. We assume that the purpose of these interactions was to make the rate equations agree with the strict 1∶1 stoichiometry of the antiport-mechanism by ensuring constant rates of overall influx and efflux [17]. Originally proposed by Portis (1983) [20], this representation has been repeatedly used in modified forms [21], [22]. The included regulatory effects cause activation and non-competitive inhibition on both sides of the membrane. However, they do not reflect true biological interactions [9] but instead serve as a means to mimic the antiport characteristics of the TPT.

**Figure 4:**
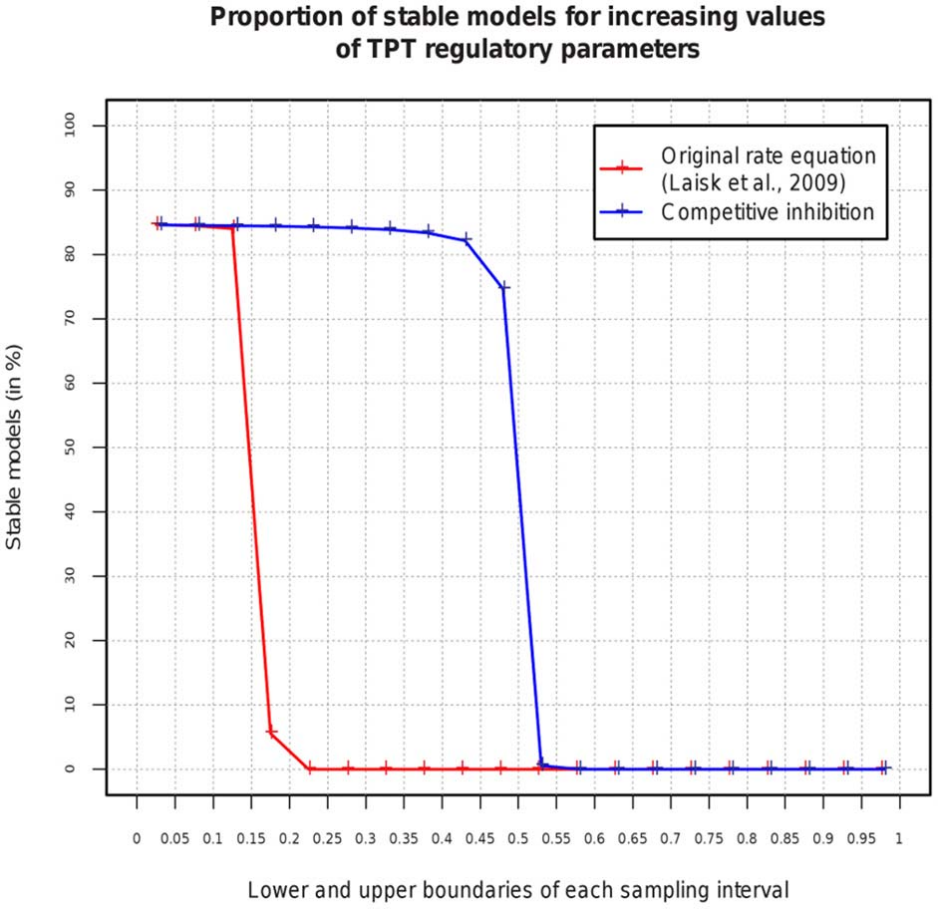
Stable steady states for increasing values of regulatory TPT parameters. Effect of increasing SK-model parameters for the triose-phosphate translocator (TPT) under different assumptions regarding regulatory mechanisms. Transporter-associated model parameters were sampled from consecutive intervals of length 0.1. For each interval, 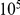 SK-models were generated.

In a structural kinetic model, these interactions are represented by eight model parameters. To analyze the implications of these eight parameters on stability we created 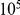 SK-models in which the TPT associated parameters were sampled from consecutive, non-overlapping intervals of length 0.1. As shown in Figure 4, the observed proportions of stable models dramatically decrease for model parameters larger than 0.15, and vanish completely for parameters exceeding 0.2. To interpret this observation, it is important to note that a low parameter value indicates that the concentration change of a specific metabolite has a low impact on the rate of a reaction. This is generally associated with high enzyme-metabolite saturation. Thus, stability seems to be favored only as long as the TPT is highly saturated by its substrates.

In contrast to these antiport-associated regulatory interactions in the original rate equation, a biologically well-known phenomenon is competitive inhibition caused by the simultaneous transport of different substrates [23]. Although this type of regulation is not included in the kinetic model, SKM enables us to analyze its potential effects on the stability of the investigated steady state. This is achieved by introducing corresponding parameters into the SK-model without the need to change the original rate equations in the kinetic model. To compare the effects of competitive inhibition on stability to those implied by the original rate equation that has been used in the kinetic model, we created SK-models with increasing intervals for the parameters associated with competitive inhibition, while the antiport-associated parameters were set to zero. The resulting proportions of stable models are shown in Figure 4. Using competitive inhibition, a steep decrease in the proportion of stable states can be observed for parameters exceeding 0.45. Thus, competitive inhibition alone would impair stability much less than the regulatory effects implied by the original rate equation which account for the antiport characteristics.

This example demonstrates how SKM can help to analyze system behavior under different assumptions about regulatory mechanisms without requiring detailed rate equations and kinetic parameters for each scenario.

### The impact of allosteric regulation on plant energy metabolism

In order to investigate the dynamic properties of the system without transporter-associated regulatory parameters, we randomly sampled SK-models of the whole system in Figure 3 while excluding the regulation of the transporter reactions. Table 1 shows the proportions of stable and unstable cases obtained from 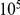 SK-models. Mean values and standard deviations were estimated by repeating the sampling procedure ten times. Stability occurred in over 84% of all cases, indicating that the design of the network is strongly targeted towards maintaining its functional state under varying physiological conditions.

**Table 1:** The impact of regulation on plant energy metabolism.

	Stable	Unstable
Original model with regulation	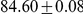	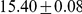
Without regulation	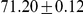	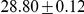
Regulation at random positions	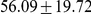	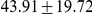
Additional regulators (BRENDA database)	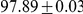	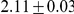

Occurrence (in %) of stable cases in 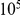 randomly generated SK-models with different types of allosteric regulators. Standard deviations were obtained by repeating the sampling procedure ten times. Original model: regulatory effects as described in the kinetic model; Without regulation: omitting all allosteric regulators; Regulation at random positions: replacement of the regulators in the original model by regulators at random positions in the network; Additional regulators: Incorporation of regulators from the BRENDA database in addition to those in the original model.

In the plant cell, the cytosolic sucrose pathway and the stromal starch pathway are subject to regulation by allosteric feedback and feed-forward effects. In the kinetic model, this is reflected by nine cases in which metabolites regulate one of the enzymes ADP-glucose pyrophosphorylase (AGPase), sucrose 6-phosphate synthase (SPS), cytosolic fructose 1,6-bisphosphatase (F16BPase) or fructose 2,6-bisphosphatase (F26BPase). In order to assess the influence of these mechanisms on stability, we sampled SK-models in which these interactions were ignored (by setting the nine corresponding SK-model parameters to zero). As shown in Table 1, the number of stable models dropped by more than 10 percentage points after removing the influence of the nine allosteric regulators in this manner. This confirms that allosteric regulation can play an important role in supporting stability of a steady state. Similar effects have also been previously observed for a network of human erythrocyte metabolism, where omitting allosteric regulation also reduced stability by 10 percentage points [4].

For comparison, we created an additional set of models in which the original regulatory mechanisms were replaced by comparable effects at random positions in the network. In contrast to the original regulation parameters, assigning parameters at random positions in the network significantly reduced the number of stable models (Table 1). This indicates that the effect on stability crucially depends on the position of regulatory interactions in the network, and not just on their frequency.

Next, we analyzed the effects of additional known regulatory interactions that were not considered in the original kinetic model. In doing so, we selected 21 regulatory mechanisms from the BRENDA database [24]. As shown in Table 1, stability was obtained in more than 97% of all SK-models, leading to an increase by more than 13 percentage points when compared to the original model. This example shows that (1) SKM enables the assessment of newly detected pathway characteristics that are not yet part of a comprehensive kinetic model, and (2) that the regulatory mechanisms assessed in this manner indeed have a high impact on the system's behavior. The latter point motivates the extension of existing kinetic models of the Calvin-Benson cycle through the incorporation of these additional regulators, because they can be expected to have significant effects on the results of simulation studies.

### Stability of the Calvin-Benson cycle as an isolated subsystem

**Figure 5:**
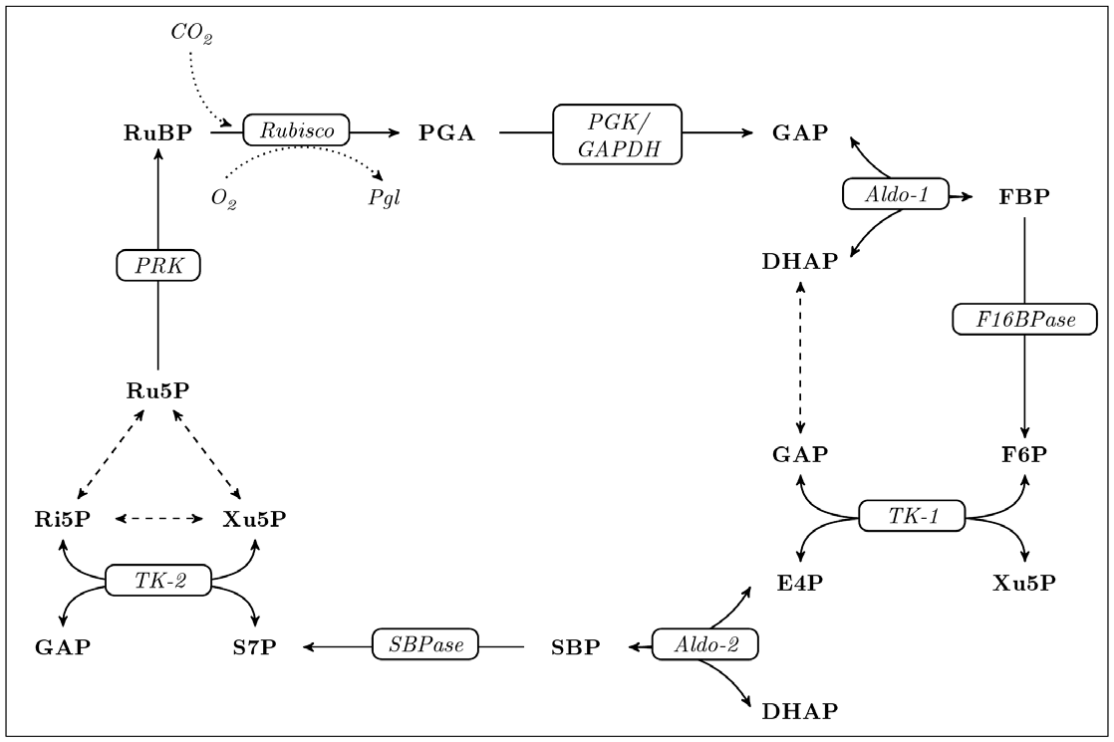
Isolated CBC subnetwork. Isolated subnetwork after restriction to metabolites which are exclusively used by the CBC. Compounds written in italics represent external substances the concentrations of which are kept constant in the model. Dotted lines indicate the reactions of cofactors. Metabolites connected by dashed lines are assumed to be in equilibrium.

In order to investigate whether instabilities can also occur in the CBC as a stand-alone system, we created 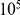 SK-models containing only those metabolites of the kinetic model that were exclusively used by the CBC (see section Methods for details). The resulting reaction network is shown in Figure 5. In doing so, we observed that 100% of these models were stable. This lack of unstable states is interesting because previous SKM-based analyses of the stability of metabolic cycles showed that, while non-autocatalytic cycles tend to be always stable, autocatalytic cycles are more sensitive and can lose their stability as a response to changes in model parameters or concentrations [12].

It is known that chloroplast metabolism depends critically on the balance of the available reducing equivalents (ATP, NADPH) [25]. However, these metabolites were excluded from the subsystem because they are used by more than just CBC enzymes. The CBC is an autocatalytic cycle with interwoven side-branches causing rearrangements of C-atoms between sugar-phosphates of different chain-length. Our results indicate that when omitting the restrictions imposed by limited availability of reducing equivalents and resources, this CBC architecture ensures stability of the investigated steady state independently of the saturation of individual enzymes.

### Identifying stability patterns by machine learning

SKM enables the identification of stable and unstable steady states for a large number of randomly generated parameter sets. When searching for model parameters with high discriminatory power between stable and unstable steady states, SKM experiments in previous research have only focused on the detection of single enzymes that played ‘key roles’ in maintaining the stability of a steady state [5]. However, experimental results indicate that metabolic control is not conducted by few key enzyme alone. Instead, changes in flux distributions can also be caused by the joint orchestration of several enzymes [7], [8]. Such orchestration of enzyme activities cannot be detected by univariate tests that simply compare the distributions of SK-model parameters between stable and unstable models. Instead, methods based on supervised machine-learning that apply either classification or regression algorithms can help identify such patterns in the model parameter space.

By using stable and unstable states as class labels, we could train classifiers based on decision trees to detect discriminating patterns in the parameter space. In doing so, we created a training data set that contained 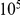 SK-models of the system in Figure 3. Each set of 87 randomly sampled model parameters served as a feature vector for classifier training. Binary class labels were introduced to indicate the presence or absence of stability associated with each parameter set. The whole procedure of model generation and machine learning is illustrated in Figure 1.

#### Classifier performance

Using 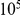 training samples, 10-fold cross validation resulted in a generalization error of 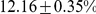. The class-dependent specificity (precision) and sensitivity (recall), summarized over all ten holdout data sets, is given in Table 2. The constructed decision trees resulted in 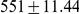 patterns per cross-validation run. Here, a pattern is a collection of conditions that restrict model parameters by upper or lower boundaries. Consequently, patterns can be understood as ensembles of coordinated criteria for enzyme-metabolite interactions responsible for maintaining or losing stability. Each pattern contained between 1 and 15 conditions. 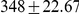 patterns provided criteria for maintaining stability (stability conditions), and 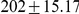 for losing it (instability conditions). All derived patterns are provided in Table S3.

**Table 2:** Classifier performance.

Class	Precision	Recall
Stable	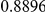	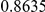
Unstable	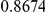	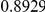

Precision and recall for each class computed by 10-fold cross-validation with 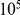 training samples. The precision of a class 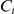 is the fraction of correct predictions compared to all predictions for this class (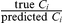); recall of a class 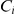 is the fraction of class members that were correctly predicted (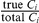).

Since our aim was to derive and investigate reliable conditions for ensuring stability or instability, we restricted our analysis to those patterns with highest accuracy. The accuracy of a pattern was assessed by its Laplace ratio, which took the number of errors (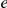) relative to the number of training samples meeting the conditions (hits 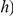 into account (see section Methods for details). The closer the Laplace value was to 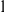, the more reliable was the pattern in defining conditions for either stability or instability. Restriction of the analysis to patterns with Laplace ratio 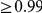 left 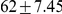 patterns with stability conditions, but only 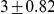 patterns with instability conditions per cross-validation run. The small occurrence frequency of reliable criteria that ensured instability might be related to a general inherent tendency towards stability in the system, which leaves only limited possibilities to cause instability in a reliable manner. These results agree with the findings in Table 1 for unbalanced data.

#### Analysis of stability conditions

**Figure 6:**
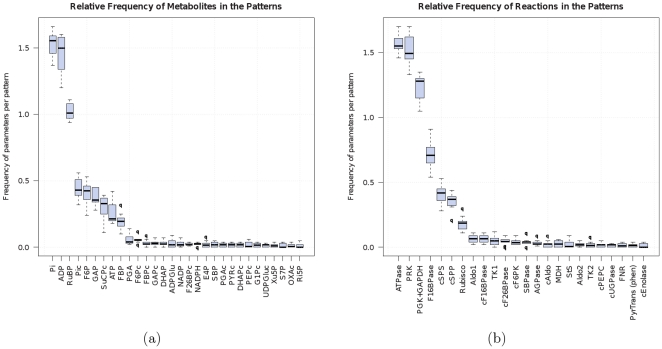
Relative frequencies of metabolites in the patterns. The boxplots show the occurrence frequencies of a) the metabolites and b) the reactions in the detected stability patterns with Laplace ratios 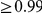 in different cross-validation runs. Cytosolic reactions begin with a lowercase ‘c’.

After restriction to Laplace ratio 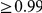, the patterns detected for the stable class each contained between 2 and 14 conditions, affecting 84 different model parameters associated with 28 enzymes and 34 metabolites. Figure 6 shows the frequencies of reactions and metabolites per pattern, obtained in each cross-validation run. The strongest control on stability was detected for the stromal metabolites Pi, adenosine diphosphate (ADP) and ribulose 1,5-bisphosphate (RuBP). The frequencies of Pi and ADP exceeded 1 due to patterns containing criteria for their interaction with more than one enzyme. The comparison of enzyme frequencies showed that the stromal reactions catalyzed by ATPase, phosphoribulokinase (PRK) and 3-phosphoglycerate kinase/glyceraldehyde 3-phosphate dehydrogenase (PGK/GAPDH) conducted most control on stability. When comparing cytosolic enzymes, the sucrose synthesis pathway enzymes SPS and sucrose 6-phosphate phosphatase (SPP) were most abundant, but still occurred only in less than half of all patterns.

In order to assess the role of assumed ‘key enzymes’ of the investigated pathways, we examined how often they appeared in the patterns. First, we analyzed the occurrence of PRK, ribulose-bisphosphate carboxylase (Rubisco), GAPDH, SBPase and stromal FBPAse, which have classically been regarded as ‘key enzymes’ of the CBC [13]. We also included the plastid aldolase (Aldo) and transketolase (TK) reactions, because *in vivo* experiments showed that these enzymes can have large impact on the rates of photosynthesis and carbohydrate accumulation [26]. Furthermore, we investigated ATPase as a key enzyme of ATP metabolism, AGPase for starch metabolism [27], and cytosolic FBPase and SPS for sucrose metabolism [23].

As shown in Tables 3 and 4, 100% of all patterns contained either conditions for model parameters associated with Rubisco, PRK, FBPase, PGK/GAPDH and SBPase. Surprisingly, conditions for sedoheptulose 1,7-bisphosphatase (SBPase) showed an average occurrence frequency of less than 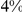 of all patterns, even if experimental results indicated that this enzyme can play an important role in controlling flux through the network [6], [28]. The AGPase reaction, which is a committed step in starch synthesis and subject to allosteric control, also appeared rarely in the patterns, whereas enzymes associated with cytosolic carbohydrate metabolism occurred in 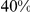 of all patterns. In contrast, the ATPase reaction was included in almost all patterns.

**Table 3:** CBC enzyme occurrences in the derived patterns.

	Assumed ‘key enzymes’ of the CBC					Further CBC enzymes	
Enzyme	Rubisco	PRK	FBPase	PGK/GAPDH	SBPase	Aldo	TK
Occurrence in stability patterns (in %)	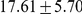	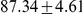	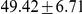	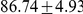	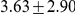	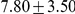	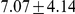
Occurrence of union set in stability patterns (in %)	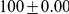					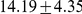	

The occurrence of parameters associated with CBC enzymes in all patterns that contain stability conditions. The enzymes are divided into assumed ‘key enzymes’, and further stromal enzymes for which high experimentally determined flux control coefficients have been reported in the literature [26]. The PGK and GAPDH reactions share common model parameters, and therefore conditions on these two reactions are joint in the patterns.

**Table 4:** Enzyme occurrences in the derived patterns (ATP, starch and sucrose metabolism).

	ATP metabolism	Starch metabolism	Sucrose metabolism	
Enzyme	ATPase	AGPase	SPS	cFBPase
Occurrence in stability patterns (in %)	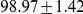	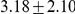	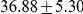	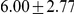
Occurrence of union set in stability patterns (in %)	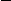	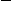	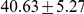	

The occurrence of parameters associated with ‘key enzymes’ of ATP, starch and sucrose metabolism in all patterns that contain stability conditions.

**Figure 7:**
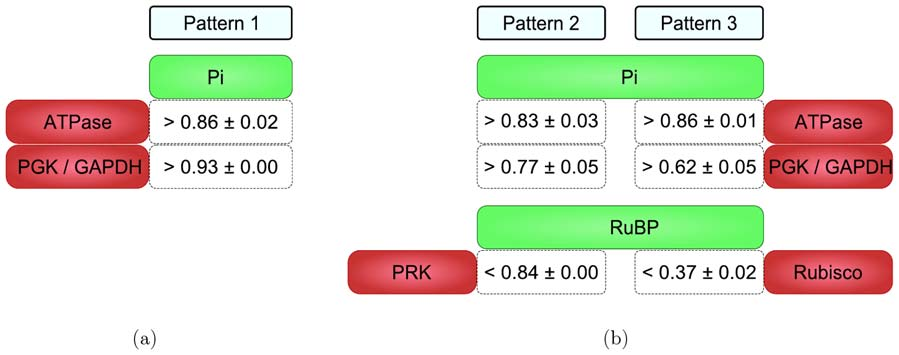
Example patterns with a) two or b) three stability conditions each. For each enzyme-metabolite pair, a threshold for the saturation 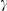 is given. Enzymes are marked in red, their reactants are marked in green. Pattern 1 exhibited an average Laplace ratio of 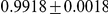 (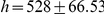, 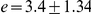). Pattern 2 affected less training samples because of its less strict threshold on the parameter associated with PGK and GAPDH but also produced more training errors and a lower Laplace value (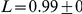 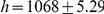, 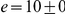). Pattern 3 affected an even larger number of hits but nevertheless, it produced fewer training errors than pattern 2. As a consequence, it exhibited the highest Laplace value of all the depicted patterns (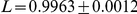, 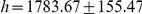, 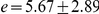).

Figure 7 shows examples of stability patterns with either two or three conditions each. The quantitative thresholds for each condition were averaged over several values obtained in different cross-validation runs. Each of the depicted patterns described high saturation of ATPase by its substrate Pi. If the saturation of GAPDH by its product Pi was sufficiently high (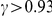), no further conditions were required (pattern 1). Because of this strict threshold, only few randomly sampled SK-models fulfilled the conditions of this pattern. Patterns with less strict thresholds that were fulfilled by larger proportions of SK-models required a third condition that either imposed upper limits on the saturation of PRK (pattern 2) or Rubisco (pattern 3) by RuBP. The positions at which the conditions of each pattern occurred in the network are illustrated in Figure S1. All three patterns indicate that it is favorable for stability to maintain high levels of Pi in the stroma, so that the corresponding enzymes are sufficiently saturated. Fast metabolization of RuBP, which reduces the saturation of PRK and Rubisco by RuBP, also favors stability.

## Discussion

We have extended SKM by a novel machine-learning based approach for determining stabilizing patterns in the parameter space using decision trees. In contrast to previous studies that used univariate tests to search for single, important parameters, it enables the search for ensembles of enzymes and metabolites ensuring stability. In algorithmic terms, these ensembles are represented by rulesets, which can be understood as ‘patterns’ of enzyme-metabolite relationships that mark the transition from stable to unstable steady states, or vice versa. This new approach allows the determination of fine-tuned interactions between combinations of several enzymes and metabolites that cannot be investigated by classical *in vivo* studies focusing only on a limited number of enzymes per experiment.

We presented and analyzed a detailed SK-model of the Calvin-Benson cycle, starch synthesis, and cytosolic carbohydrate metabolism. In total, it comprised 35 metabolites, 29 reactions and 87 model parameters. Using a Monte Carlo approach, we showed the effect of different kinds of regulation for the triose phosphate transporter. Our findings highlight the importance of choosing appropriate rate equations when modeling transport processes that serve as bottlenecks of flux between compartments. We also investigated the effects of metabolic regulation and showed that it can significantly facilitate to maintain stability. However, we also showed that regulatory interactions have to occur at very specific positions in the network to cause this effect. Even without allosteric regulation the system was stable in more than 70% of all randomly created models. This trend towards stability was confirmed when we investigated a subsystem restricted to Calvin-Benson cycle metabolites only.

The machine-learning detected patterns contained up to 15 conditions, indicating that it is often insufficient to change the saturation of only one enzyme in order to induce an effect. Rather, a change in dynamic properties often requires concerted alterations in several model parameters. We investigated the detected patterns with respect to their prediction accuracy, discovering more patterns that reliably ensured stability than patterns ensuring instability. We found that a criterion for ensuring stability is a sufficiently high saturation of ATPase by its substrate Pi. This makes a drop of stromal phosphate the most likely threat to stability.

The patterns that reliably ensured stability all contained conditions for at least one of the enzymes Rubisco, FBPase, PGK, GAPDH, PRK or SBPase. In contrast, aldolase or transketolase occurred much less frequently in the detected patterns, indicating that they play less important roles in maintaining the stability of the investigated steady state. However, in *in vivo* experiments they exhibited comparably strong influence on the flux through the pathway [26], [28]. This contradictory behavior can be explained when comparing the variables measured *in vivo* to the criteria used in this study. In the mentioned experiments, flux control coefficients were computed to assess the influence of an enzyme on carbon fixation or carbohydrate accumulation. Consequently, they described the impact of an enzyme on the flux through the system from source to sink. In contrast, using SKM we could analyze the role of enzymes in controlling the dynamic behavior of the system in a steady state under the assumption of constant influx and efflux. Consequently, the detected mechanisms helped to maintain constant internal fluxes through the cycle in a slightly perturbed system, requiring that both influx and efflux remain unchanged.

The abundance of CBC enzymes in the patterns highlighted the importance of the fine-tuned control of fluxes through the CBC in order to ensure stability of the entire network. However, none of the CBC ‘key enzymes’ appeared in 100% of the detected patterns. This shows that none of these enzymes were sufficient to ensure stability by themselves without also taking into account the activity of other enzymes.

Interactions between enzymes and metabolites can only be assessed by SKM if they are incorporated into the fixed network structure. Consequently, all metabolites or enzymes associated with model parameters must be represented by separate rows or columns of the stoichiometric matrix. We used a kinetic model as a reference for the model structure [17]. This model was chosen because of its capability to well reproduce experimentally measured data. However, there are some limitations to this model. For example, the low abundance of AGPase in the patterns could be caused by the simplified representation of starch synthesis in the model. The detection of starch-associated conditions might require a more refined representation of the starch pathway, possibly including starch degradation [27], [29]–[31]. Furthermore, 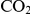 could not be represented by SK-model parameters because its concentration depended predominantly on the atmospheric conditions, and not on enzymatic reactions included in the stoichiometric matrix. Because changes in ambient 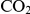 can strongly affect flux control coefficients of CBC enzymes on photosynthesis [26], the incorporation of 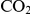 in the SK-model could potentially refine the patterns detected by the presented machine learning approach. Alternatively, the impact of varying 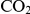 saturation on stability could also be systematically investigated using the same SK-model, but different steady states measured under varying 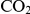 concentrations.

In the presented work, we applied our method to steady state data that represents the operating point in a C3 plant under atmospheric conditions (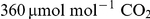) and high light (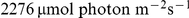). Using this particular steady state, we demonstrated the application of our combined SKM and machine learning approach, and we showed how to reveal the regulatory patterns ensuring its stability. However, if the aim is to gain a full understanding of the patterns that can emerge in the CBC, it would also be necessary to investigate alternative steady states under varying physiological conditions. For example, a comparison of stabilizing mechanisms could provide hints about the robustness of specific types of network regulation.

The CBC is a well-studied system for which detailed kinetic models are already available, even though there is still an ongoing debate about their quality and predictive power [9], [10]. However, an advantage of SKM is that it does not rely on such detailed information in order to yield meaningful results. Under the prerequisite that accurate measurements of steady state concentrations and fluxes are available, the proposed method also enables the detection of stabilizing mechanisms in networks for which comprehensive kinetic models have not yet been developed. We showed in this work that incorporation of known allosteric interactions can significantly increase the tendency towards stability. Under the hypothesis that regulatory mechanisms can generally be expected to improve stability of a steady state, SKM could potentionally also be applied to detect unknown allosteric effects in such networks. In doing so, randomly inserted regulatory effects could be assessed by SKM, and those mechanisms that are most beneficial for stability could be selected and tested experimentally.

## Methods

### Algorithmic background

#### Stability analysis of metabolic systems

A metabolic system can be formulated as an ordinary differential equation system that describes the time-dependent changes in metabolite concentrations:
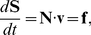
(1)where the vector 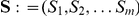 contains the concentration of 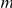 metabolites, the vector 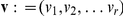 contains the velocities of 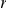 reactions, and the stoichiometric matrix 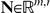 contains the molecularities of substrates and products in each reaction, which are stored in its elements 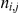 [32]. The time-dependent changes in each concentration are then summarized in the vector 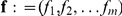. In metabolic networks, the reaction rates 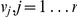 depend non-linearly on the metabolite concentrations so that 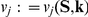. The parameter vector 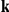 contains additional kinetic information like maximum velocities or binding affinities of the enzymes.

A steady state is defined as a point 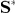 in the state space where 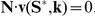 Hence, no net changes in the concentrations can occur, and the rate of production equals the rate of consumption for each metabolite. Consequently, a system can only leave a steady state in response to changes caused by external factors, for example affecting the flux into the system or variations in enzyme concentrations.

The response of the system to small perturbations depends on its stability properties. When the steady state is stable, a coordinated system response enables the return of concentrations and fluxes to the same values as prior to the perturbation. If the steady state is unstable, such a return is not supported. The stability of a steady state of system (1) can be assessed by the system's Jacobian matrix 
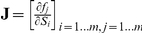
 evaluated in the steady state. The Jacobian matrix contains the partial derivatives of the metabolite turnover rates with respect to all substrates, products and regulators that take part in the reactions. If the largest real part of its eigenvalues is negative, changes evoked by perturbations diminish over time and the steady state is stable [32].

#### The principles of structural kinetic modeling

Computation of the Jacobian matrix of a metabolic system at an arbitrary point in the state space requires knowledge of all enzyme kinetic rate laws and kinetic parameters describing the reactions in the system. In a steady state with concentrations 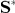 and fluxes 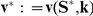, however, SKM enables the computation of the Jacobian matrix 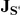 without requiring this knowledge. Instead, the Jacobian matrix can be derived from a set of model parameters 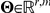, the stoichiometric matrix 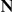, and the steady state information according to the formula
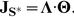
(2)Here 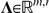 is a matrix of normalized stoichiometric coefficients with elements 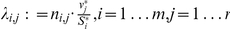. The matrix 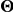 contains the model parameters 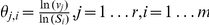, which describe the relative influence of each metabolite on each reaction rate in the steady state (see Document S1 for details).

While the stoichiometry, the steady state concentrations and the fluxes are experimentally accessible, the model parameters are often unknown in practice. However, they can be sampled in a Monte Carlo approach, which enables the creation of a large number of models followed by the exploration of the parameter space to detect regions associated with stability or instability. Sampling intervals are derived from the type of kinetics the reaction is expected to follow (for example Michaelis-Menten or Hill kinetics).

In total, the SKM approach can be summarized by the following steps: (1) normalization of the stoichiometric matrix with respect to steady state fluxes and concentrations; (2) random sampling of the model parameters from intervals chosen according to the type of kinetics that is assumed for the reaction (see Document S1 for details); (3) computation and evaluation of the Jacobian matrix for each sampled parameter set.

#### Deriving a measure for enzyme-metabolite saturation from the SK-model parameters

The closer the absolute value of a parameter 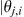 is to the upper limit of its sampling interval (for example 1 for Michaelis-Menten kinetics), the less the enzyme catalyzing the reaction 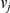 is saturated with its metabolite 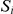. Therefore we define by
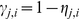
(3)the actual saturation implied by the model parameters 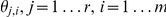. Here, 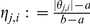 is the absolute value of 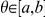 normalized to the interval 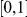.

#### Matlab implementation of structural kinetic modeling

We developed a MATLAB algorithm for generic creation and evaluation of structural kinetic models for arbitrary pathways. Necessary input information are the stoichiometric matrix of the pathway, the steady state concentrations and fluxes and the limits of the sampling intervals for each model parameter. The sampling procedure can be modified by user-defined options, for example to omit regulatory interactions or to exclude individual metabolites from the analysis. Automatic evaluation routines are provided to assist analysis of the produced models, for example by plotting the eigenvalue distributions, computing the proportions of stable and unstable models, or producing training data as input for the C5.0 classification algorithm [33]. Currently, the code is available on request, a manuscript introducing the algorithm in greater detail is in preparation.

### Building the structural kinetic model of the Calvin-Benson cycle

#### Computation of steady state data

Steady state concentrations and fluxes were computed using the kinetic model by Laisk et al. (2009) [17]. The model was slightly modified compared to the original version by introducing separate differential equations for dependent metabolites and for pooled metabolites. After implementing the model in MATLAB, we performed numerical integration using the ode15 s solver starting from the initial values which were provided by the authors in their original PASCAL implementation of the model. Integration was performed over 1000 seconds until all concentration changes were below 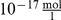.

#### Deriving model parameter intervals for enzymatic reactions

We derived interval boundaries for most reactions based on their rate laws in the kinetic model. Some enzyme-catalyzed reactions were represented by simplified rate equations in the kinetic model, for example by using mass action kinetics instead of Michaelis-Menten kinetics. This would correspond to a situation where the model parameters are set to 1, and the enzyme is barely saturated. In the SK-models, we treated these reactions as if following Michaelis-Menten kinetics in order to enable investigation of a broader range of saturation values (see the Document S1 for details).

#### SK-model parameters for the triose-phosphate translocator (TPT)

Regulatory mechanisms in the transport-associated rate equations used in the kinetic model were represented by four positive (activating), and four negative (inhibiting) parameters, each accounting for an interaction on the stromal, as well as on the cytosolic side of the membrane. The four positive parameters describe antiport-induced activation of import reactions by stromal species, and of export reactions by cytosolic species. The four negative parameters describe the inhibition of export reactions by their stromal substrates, and of import reactions by their cytosolic substrates. In order to assess the influence of these interactions on stability, we created 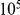 SK-models by randomly sampling all 8 parameters from predefined intervals with increasing upper and lower boundaries. To assess the effects of competitive inhibition, we repeated the sampling procedure, this time only regarding those parameters that described inhibition of export by stromal species or inhibition of import by cytosolic species (see Document S1 for details).

### Analyzing the structural kinetic model of the Calvin-Benson cycle

#### Assessing the influence of allosteric regulation in the kinetic model

In the kinetic model, the enzymes AGPase, cytosolic FBPase, as well the reactions for synthesis and degradation of cytosolic F26BP are allosterically regulated by sugar phosphates or inorganic phosphate. In total, we obtained 6 positive and 3 negative model parameters. A comprehensive list of all regulatory parameters and their sampling intervals is given in Table S2. In order to determine the influence of these interactions, we created a set of 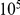 SK-models were these parameters were set to zero. Based on the eigenvalues of the resulting Jacobian matrix we then determined the number of stable models. Standard deviations were obtained by repeating this procedure ten times. For validation, we created SK-models with allosteric regulation at random positions in the network. In doing so, we randomly selected 9 positions in the matrix 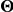 which we occupied by allosteric regulators. Using this set of regulatory parameters, we randomly sampled 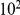 SK-models. This procedure of randomly assigning positions for regulatory interactions and sampling SK-models using these interactions was repeated 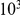 times. Mean values and standard deviations were computed from the proportions of stable models obtained by each repetition.

#### Incorporation of additional regulatory interactions from the BRENDA database

In order to assess the impact of experimentally obtained activators and inhibitors of the investigated system, we included model parameters for regulatory interactions that were reported in the BRENDA database for either of the organisms *Arabidopsis thaliana*, *Nicotiana tabacum*, and *Spinacia oleracea*. In total, we obtained 14 additional model parameters describing activating effects, as well as 7 parameters for inhibitory effects. A detailed list of these parameters and their sampling intervals is given in Table S2.

#### Analysing a subsystem of Calvin-Benson cycle metabolites

In order to restrict the analyses on a subsystem that contained only CBC metabolites, we removed all rows for non-CBC metabolites from the stoichiometric matrix 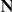. The resulting matrix 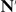 still fulfilled the steady-state requirement 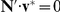 and therefore allowed the construction of SK-models restricted to the remaining metabolites and the reactions they involved. We constructed SK-models of the CBC as a stand-alone system by constraining the stoichiometric matrix to those stromal compounds that were exclusively involved in CBC rations. These metabolites comprised PGA, GAP, DHAP, erythrose 4-phosphate (E4P), ribose 5-phosphate (Ri5P), ribulose 5-phosphate (Ru5P), xylulose 5-phosphate (Xu5P), ribulose 1,5-bisphosphate (RuBP), fructose 6-phosphate (F6P), fructose 1,6-bisphosphate (FBP), sedoheptulose 7-phosphate (S7P) and sedoheptulose 1,7-bisphosphate (SBP).

### Classification by decision trees

Using stability as class labels, we trained decision trees in order to detect discriminating patterns in the parameter space. In doing so, we first created a training data set by randomly sampling 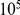 SK-models of the system. Parameters associated with transporter regulation were omitted so that the analysis focused on the effects of the remaining 87 model parameters. Each set of model parameters served as a feature vector for classifier training, and the presence or absence of stability served as a binary class label. Before training, the data set was balanced by repeatedly sampling model parameters until equal numbers of stable and unstable cases were obtained.

Training was performed using the C5.0 algorithm version 2.01 [33], a commercial version of the C4.5 algorithm [34], with increased speed and memory efficiency that makes it well applicable for large numbers of training samples. One interesting feature of this algorithm is the possibility to create ‘rulesets’ that summarize the derived conditions for each class in an easily interpretable manner. In contrast to the classical decision tree structure, where the abundance of a feature depends on its position in the tree (for example, the feature in the root is always used), features in rulesets can be mutually exclusive. This motivates their usage for the given task in which we aim at finding diverse combinations of features important for different dynamic properties. The discovered rulesets describe patterns for combinations of enzyme-metabolite interactions.

After training, the prediction performance of each of the obtained rulesets was assessed by the Laplace ratio
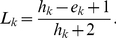
(4)Here, 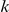 is the ruleset index, 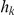 is the number of training samples meeting the conditions given by the 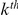 ruleset (hits), and 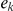 is the number of samples with opposite class label to that indicated by the ruleset (errors) [33]. The derived rulesets and their properties are given in Table S3. Since our aim was to derive reliable conditions for stability and instability, we selected only those rules with Laplace ratio 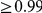 for further analyses.

## Supporting Information

Figure S1 **Network plot showing the localization of the patterns which have been introduced in** **Figure 7** **of the Results section.** Blue circles highlight conditions in pattern 1, red circles indicate conditions in pattern 2, and green circles show conditions in pattern 3.
(PDF)

Table S1 **Table of the model components. Spreadsheet 1 (‘Compounds’): model compounds which are depicted in** **Figure 3** **and in Figure S1.** The left table contains those compounds which served as state variables in the kinetic model, and are therefore associated with SK-model parameters. The right table contains those compounds which were treated as external metabolites with constant concentrations in the kinetic model, and therefore do not yield SK-model parameters. Spreadsheet 2 (‘Reactions’): reactions associated with SK-model parameters. For each reaction, we provide the abbreviation used in this text, the KEGG-ID, the stoichiometry applied in the SK-model as well as activating or inhibiting compounds.
(ODS)

Table S2 **Table of sampling intervals for the SK-model parameters.** Spreadsheet 1 (‘Regulatory parameters’): network positions and sampling intervals of the SK-model parameters associated with regulatory interactions. Spreadsheet 2 (‘TPT Parameters’): sampling intervals and dependencies of the SK-model parameters associated with the Triose-Posphate/Phosphate Translocator (TPT). Spreadsheet 3 (‘Sampling interval modifications’): modifications of the original sampling intervals (derived from the original rate equations in the kinetic model) used to create the SK-models.
(ODS)

Table S3
**Machine-learning derived rulesets.** The whole set of rulesets obtained by C5.0-training with 10-fold cross-validation. Spreadsheet 1 (‘Ruleset information’): information associated with each ruleset (ID, number of detected conditions, assigned class, Laplace ratio etc.). Spreadsheet 2 (‘Ruleset conditions’): detailed list of the conditions contained in each ruleset. For each condition, the reaction-metabolite pair associated with the corresponding SK-model parameter, as well as the type of their interaction (product, substrate, inhibitor or activator) is given. The corresponding threshold is described in terms of the model parameter value (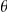), and the saturation value (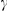) which has been computed according to equation (3).(ODS)

Document S1 **Additional background information about structural kinetic modeling, together with further information about the employed model.**
(PDF)
