# Supplementary figures and images for: Systematic Analysis of Stability Patterns in Plant Primary Metabolism
Source: PLoS One. 2012 Apr 13;7(4):e34686. doi: 10.1371/journal.pone.0034686 (PMC3326025; doi:10.1371/journal.pone.0034686)

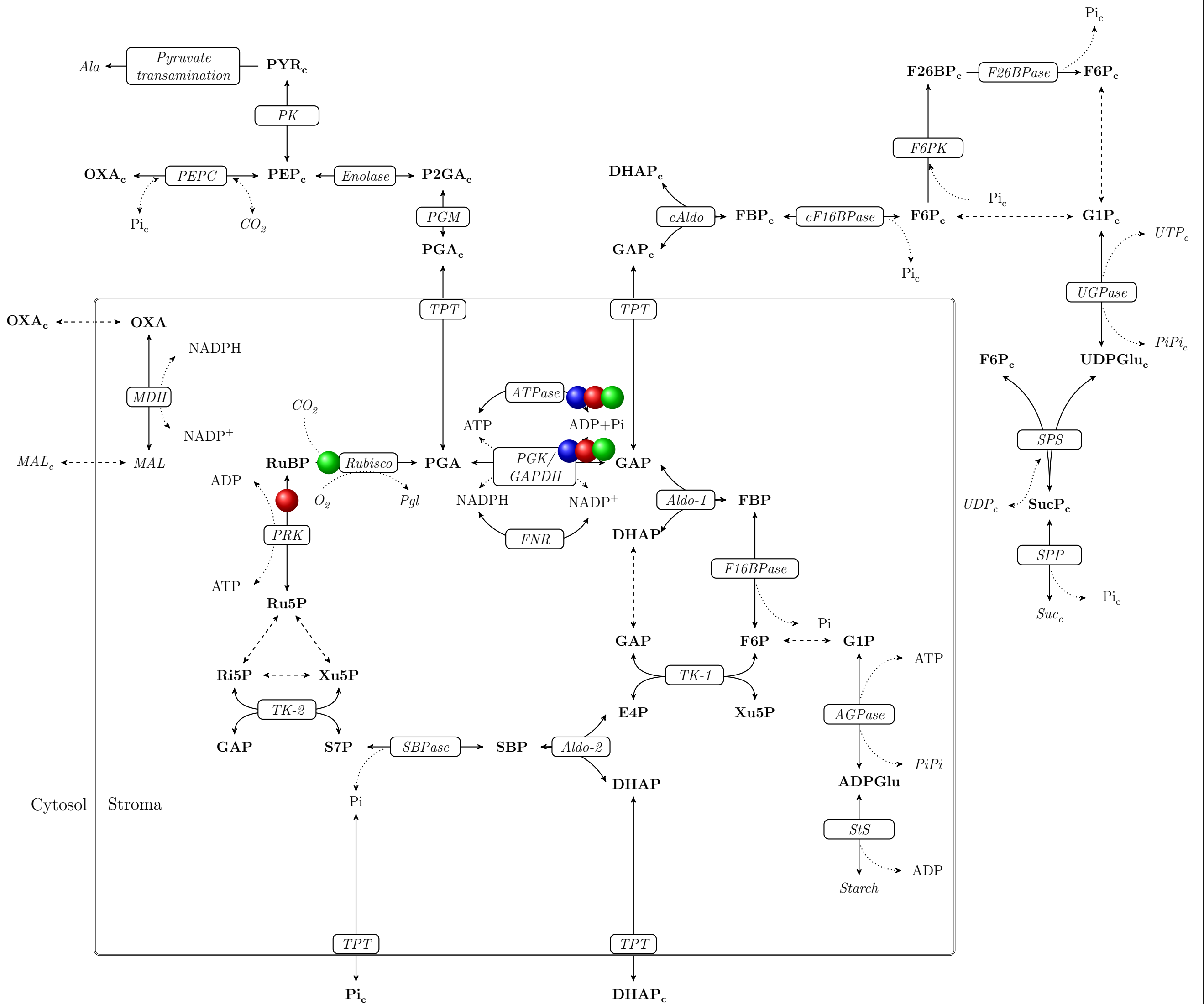

Supplement: Figure S1 — Network plot showing the localization of the patterns which have been introduced in Figure 7 of the Results section. Blue circles highlight conditions in pattern 1, red circles indicate conditions in pattern 2, and green circles show conditions in pattern 3. (PDF) [file pone.0034686.s001.pdf]
