# Supplementary material for: Systematic Analysis of Stability Patterns in Plant Primary Metabolism
Source: PLoS One. 2012 Apr 13;7(4):e34686. doi: 10.1371/journal.pone.0034686 (PMC3326025; doi:10.1371/journal.pone.0034686)
Supplement: Document S1 — Additional background information about structural kinetic modeling, together with further information about the employed model. (PDF) [file pone.0034686.s005.pdf]

Supplementary Information

1 Parameterization of the Jacobian matrix in a steady state

The Jacobian matrix $\mathbf{J} \in \mathbb{R}^{m,m}$ of a metabolic system can be decomposed into the stoichiometric matrix $\mathbf{N} \in \mathbb{R}^{m,r}$ and the gradient matrix $\mathbf{G} \in \mathbb{R}^{r,m}$ according to

$$\mathbf{J} = \mathbf{N} \cdot \mathbf{G}. \tag{1}$$

$\mathbf{N}$ describes the stoichiometry of each reaction, which is specific for the investigated pathway and does not depend on the kinetics of its reactions. In contrast, $\mathbf{G}$ contains the partial derivatives of the reaction rates, defined as $g_{j,i} = \frac{\partial v_j}{\partial S_i}$. This dynamic information depends solely on the kinetic rate laws employed in the model [1].

The gradient matrix in a steady state $\{\mathbf{S}^*, \mathbf{v}^*\}$ can be further decomposed according to

$$\mathbf{G}_{|\mathbf{S}=\mathbf{S}^*} = \mathbf{D}_{v^*} \cdot \mathbf{\Theta} \cdot \mathbf{D}_{S^*}^{-1}. \tag{2}$$

Here, $\mathbf{D}_{v^*}$ and $\mathbf{D}_{S^*}$ are diagonal matrices with the steady state fluxes or concentrations on the diagonal, and $\mathbf{\Theta}$ contains the logarithmic partial derivatives $\theta_{j,i} = \frac{\partial \ln(v_j)}{\partial \ln(S_i)}_{|S_i=S_i^*}$ [2]. Its elements can be rewritten as

$$\theta_{j,i} = \frac{\partial \ln(v_j)}{\partial \ln(S_i)}_{|S_i=S_i^*} = \frac{\partial \mu_j}{\partial x_i}_{|x_i=1}, \tag{3}$$

with $x_i(t) := \frac{S_i(t)}{S_i^*}$ and $\mu_j(x_1, x_2, \ldots x_m) := \frac{v_j(S_1,S_2,\ldots,S_m)}{v_j(S_1^*,S_2^*,\ldots S_m^*)} = \frac{v_j(S_1^* x_1, S_2^* x_2, \ldots S_m^* x_m)}{v_j^*}$ [3].

Combining equations (1) and (2) provides

$$\mathbf{J}_{|\mathbf{S}=\mathbf{S}^*} = \underbrace{\mathbf{D}_{S^*}^{-1} \cdot \mathbf{N} \cdot \mathbf{D}_{v^*}}_{=:\mathbf{\Lambda}} \cdot \mathbf{\Theta}. \tag{4}$$

The matrix $\mathbf{\Lambda}$ is a normalized version of $\mathbf{N}$ and specific for the investigated steady state. It can be understood as the stoichiometric matrix of a normalized system $\frac{d\mathbf{x}}{dt} = \mathbf{\Lambda} \cdot \boldsymbol{\mu}$. This system describes the relative movement of the trajectories with respect to the steady state. The matrix $\mathbf{\Theta}$ then serves as a gradient matrix of this system. In other words, we replace the original system which describes the absolute concentrations curves $\mathbf{S}(t)$ by a new system where the state variables $\mathbf{x}(t)$ describe the course of these concentrations relative to the predefined steady state. This transformation facilitates the computation of the Jacobian matrix in the steady state, which now depends on a set of model parameters $\mathbf{\Theta}$ [4].

However, the elements of the parameter matrix $\mathbf{\Theta}$ are often not experimentally accessible. Instead, they are randomly sampled in a Monte Carlo approach from uniform distributions over predefined intervals. The interval boundaries are chosen according to the type of kinetics employed by an enzyme. Details about the computational derivation of these intervals can be found in the supplementary information of Steuer et al. [3]. Table 1 provides an overview of the sampling interval boundaries for various enzyme kinetics.

Interaction type	Sampling interval
Michaelis-Menten substrate	$[0, 1]$
Michaelis-Menten product	$[-1, 0]$
Allosteric activation with Hill coefficient n	$[0, n]$
Allosteric inhibition with Hill coefficient n	$[-n, 0]$

Table 1: Sampling intervals for SK-model parameters representing different types of enzyme-metabolite interactions.

2 Constructing the structural kinetic model of the Calvin-Benson cycle

The construction of a structural kinetic model of a metabolic system requires the computation of the matrix $\mathbf{\Lambda}$ which is derived by normalizing the stoichiometric matrix $\mathbf{N}$ with respect to the steady state concentrations $\mathbf{S}^*$ and fluxes $\mathbf{v}^*$. An additional requirement is the parameter matrix $\mathbf{\Theta}$ which contains the SK-model parameters necessary to compute the Jacobian matrix. In order to determine the sampling intervals for these parameters, we used the enzyme-metabolite interactions given by the kinetic model of Laisk et al. (2009) [5] as a reference. In the following, we describe how we constructed the SK-model based on this information. In some cases, the information obtained from the kinetic model was slightly altered to enable more detailed evaluation of different dynamic scenarios.

Structural information: the normalized matrix $\mathbf{\Lambda}$

Taking the individual steps of the Rubisco reaction into account, the original kinetic model included 35 reactions. A detailed overview of the individual reaction mechanisms, reactants and regulators of each reaction is given in Table 1. Each reaction was separated into its forward and backward reaction, so that the stoichiometric matrix $\mathbf{N}$ contained 70 columns.

In the original kinetic model, 41 concentration values (35 metabolites, 6 enzyme concentrations) were computed in each integration step. 24 of these compounds are modeled directly by differential equations. 13 were computed from five different metabolite pools that described metabolites in equilibrium ({GAP, DHAP}, {Ri5P, Ru5P, Xu5P}, {G1P, G6P, F6P}, {$\mathrm{GAP_c}$, $\mathrm{DHAP_c}$}, {$\mathrm{G1P_c}$, $\mathrm{G6P_c}$, $\mathrm{F6P_c}$}). Each metabolite pool was modeled by a differential equation, and concentrations of the individual compounds in the pools were calculated in each integration step based on their equilibrium constants. Four compounds depended directly on other concentrations and were computed internally in each integration step (ADP, NADP, Ef, $\mathrm{Pi_c}$).

In order to be able to assign SK-model parameters to each of the 13 compounds in the metabolites pools, we derived separate differential equations and included them as separate rows in the stoichiometric matrix. Additionally, we derived four differential equations for those metabolites whose concentrations depended directly on other compounds. In total, these modifications increased the number of state variables for which SK-model parameters could be derived from 24 to 41.

Because this study focused on interactions taking place in the CBC and downstream reactions, we omitted photosynthetic light reaction so that the rates of photosynthesis and NADPH regeneration were assumed to be constant. PGK and GAPDH shared the same column in the stoichiometric matrix because of their joint representation by the same rate law in the kinetic model. A schematic overview of the reactions and metabolites included in the model is given in Figure 3.

Using steady state concentrations $\mathbf{S}^*$ and fluxes $\mathbf{v}^*$ computed by the kinetic model, the elements of matrix $\mathbf{\Lambda}$ were derived by $\lambda_{i,j} = n_{i,j} \cdot \frac{v_j^*}{S_i^*}, \quad i = 1 \ldots 41,\ j = 1 \ldots 70.$

Kinetic information: the model parameter matrix $\mathbf{\Theta}$

Excluding transporter associated parameters, the SK-models of the system in Figure 3 contained 87 model parameters. Some changes were made to the sampling intervals derived from the original kinetic model:

1. Instead of describing individual steps of the Rubisco reaction by mass action kinetics, we sampled the saturation parameters for its substrates and products from the interval $[0, 1]$ in order to investigate the role of the corresponding enzyme-metabolite interactions for stability.

2. NADPH and NADP were included as substrate and product of the joint reaction PGK/GAPDH.

3. Parameters for some reactions which for simplicity had been modeled by mass-action kinetics (for example F26BP synthesis and degradation), were instead treated as Michaelis-Menten parameters and sampled from the interval $[0, 1]$.

Transporter-associated parameters derived from the original rate equations

The rate equation used in the kinetic model to describe the triosephosphate / phosphate translocator (TPT) is based on mass action kinetics, but is modified to make it agree more closely with the antiport-mechanism.

Using only mass action kinetics, the rate of import and export would be proportional to the concentrations of the reactants on both sides of the membrane. However, this would not reflect the true biology, because the antiport mechanism couples both rates to each other. The kinetic model therefore uses rate equations that include several regulatory mechanisms in order to account for the antiport mechanisms. Further details about these equations are given by Portis (1983) [6]. They are also used for modeling transport processes in other kinetic models of the CBC [7, 8].

Computation of the derivatives of the normalized system revealed that the model parameter which described the effect of a stromal metabolite on efflux processes had the same value as the parameter which described the effect of the same cytosolic metabolite on influx processes and vice versa. Consequently, it was sufficient to employ 8 model parameters in order to describe all regulatory influences of metabolites on TPT-mediated transport processes in the kinetic model. Table 2 summarizes the necessary parameters and their possible interpretation. All parameters were sampled from the interval $[0, 1]$ (activatory effects) or $[-1, 0]$ (inhibitory effects). The position of these parameters in the matrix $\mathbf{\Theta}$ are given in Table S2.

Parameter	Regulatory effects on the stromal side	Regulatory effects on the cytosolic side
θ_{TPT1}	Inhibition of export processes by stromal PGA	Inhibition of import processes by cytosolic PGA
θ_{TPT2}	Inhibition of export processes by stromal GAP	Inhibition of import processes by cytosolic GAP
θ_{TPT3}	Inhibition of export processes by stromal DHAP	Inhibition of import processes by cytosolic DHAP
θ_{TPT4}	Inhibition of export processes by stromal Pi	Inhibition of import processes by cytosolic Pi
θ_{TPT5}	Activation of import processes by stromal PGA	Activation of export processes by cytosolic PGA
θ_{TPT6}	Activation of import processes by stromal GAP	Activation of export processes by cytosolic GAP
θ_{TPT7}	Activation of import processes by stromal DHAP	Activation of export processes by cytosolic DHAP
θ_{TPT8}	Activation of import processes by stromal Pi	Activation of export processes by cytosolic Pi

Table 2: Model parameters describing phosphate translocator regulation (original rate equation including antiport constraints).

Competitive inhibition of transport processes

In order to investigate the effects of competitive inhibition instead of the regulatory effects which intend to mimic antiport constraints, we introduced 8 model parameters that represented inhibition of efflux of each metabolite by competing stromal compounds, as well as inhibition of influx of each metabolite by competing cytosolic compounds. All parameters are listed in Table 3 and explained in greater detail in Table S2.

Parameter	Regulatory effects
θ^*_{TPT1}	Inhibition of PGA and Pi export by stromal GAP
θ^*_{TPT2}	Inhibition of PGA and Pi export by stromal DHAP
θ^*_{TPT3}	Inhibition of PGA, GAP and DHAP export by stromal Pi
θ^*_{TPT4}	Inhibition of Pi, GAP and DHAP export by stromal PGA
θ^*_{TPT5}	Inhibition of PGA and Pi import by cytosolic GAP
θ^*_{TPT6}	Inhibition of PGA and Pi import by cytosolic DHAP
θ^*_{TPT7}	Inhibition of PGA, GAP and DHAP import by cytosolic Pi
θ^*_{TPT8}	Inhibition of Pi, GAP and DHAP import by cytosolic PGA

Table 3: Model parameters describing phosphate translocator regulation (competitive inhibition only).

References

[1] Jamshidi N, Palsson BO (2008) Formulating genome-scale kinetic models in the post-genome era. Molecular Systems Biology 4: 171.

[2] Steuer R (2011) Exploring the dynamics of large-scale biochemical networks: A computational perspective. The Open Bioinformatics Journal 5: 4–15.

[3] Steuer R, Gross T, Selbig J, Blasius B (2006) Structural kinetic modeling of metabolic networks. Proceedings of the National Academy of Sciences of the United States of America 103: 11868–11873.

[4] Gross T, Feudel U (2006) Generalized models as a universal approach to the analysis of nonlinear dynamical systems. Physical Review E, Statistical, Nonlinear, and Soft Matter Physics 73: 016205.

[5] Laisk A, Eichelmann H, Oja V (2009) Leaf C3 photosynthesis *in silico*: Integrated carbon/nitrogen metabolism. Dordrecht, volume 29. pp. 295–322.

[6] Portis AR (1983) Analysis of the role of the phosphate translocator and external metabolites in steady-state chloroplast photosynthesis. Plant Physiology 71: 936–943.

[7] Pettersson G, Ryde-Pettersson U (1988) A mathematical model of the Calvin photosynthesis cycle. European Journal of Biochemistry 175: 661–672.

[8] Zhu X, de Sturler E, Long SP (2007) Optimizing the distribution of resources between enzymes of carbon metabolism can dramatically increase photosynthetic rate: a numerical simulation using an evolutionary algorithm. Plant Physiology 145: 513–526.
